# The Reassuring Absence of Acute Stress Effects on IQ Test Performance

**DOI:** 10.3390/jintelligence13100131

**Published:** 2025-10-19

**Authors:** Osman Akan, Mustafa Yildirim, Oliver T. Wolf

**Affiliations:** Department of Cognitive Psychology, Institute of Cognitive Neuroscience, Faculty of Psychology, Ruhr University Bochum, 44780 Bochum, Germany; mustafa.yildirim@rub.de (M.Y.); oliver.t.wolf@rub.de (O.T.W.)

**Keywords:** acute stress, test anxiety, intelligence, IQ test

## Abstract

Acute stress impairs executive functions, and these higher-order cognitive processes are often positively associated with intelligence. Even though intelligence is generally stable over time, performance in an intelligence test can be influenced by a variety of factors, including psychological processes like motivation or attention. For instance, test anxiety has been shown to correlate with individual differences in intelligence test performance, and theoretical accounts exist for causality in both directions. However, the potential impact of acute stress before or during an intelligence test remains elusive. Here, in a research context, we investigated the effects of test anxiety and acute stress as well as their interaction on performance in the short version of the Intelligence Structure Test 2000 in its German version (I-S-T 2000 R). Forty male participants completed two sessions scheduled 28 days apart, with the order counterbalanced across participants. In both sessions, participants underwent either the socially evaluated cold-pressor test (SECPT) or a non-stressful control procedure, followed by administration of I-S-T 2000 R (parallelized versions on both days). The SECPT is a widely used laboratory paradigm that elicits a stress response through the combination of psychosocial and physical components. Trait test anxiety scores were obtained via the German Test Anxiety Inventory (TAI-G). Stress induction was successful as indicated by physiological and subjective markers, including salivary cortisol concentrations. We applied linear mixed models to investigate the effects of acute stress (elicited by our stress manipulation) and test anxiety on the intelligence quotient (IQ). The analysis revealed that neither factor had a significant effect, nor was there a significant interaction between them. Consistent with these findings, Bayesian analyses provided evidence supporting the absence of these effects. Notably, IQ scores increased significantly from the first to the second testing day. These results suggest that neither test anxiety nor stress is significantly impacting intelligence test performance. However, improvements due to repeated testing call for caution, both in scientific and clinical settings.

## 1. Introduction

Imagine you are about to take a test that will determine the trajectory of your future career. As the test approaches, your heart beats faster, your palms sweat, and your pupils dilate, a cascade of visible symptoms reflecting your body’s response to stress. Then some hormones, such as (nor)adrenaline and cortisol, are released into your bloodstream to prepare you for the demands of the upcoming challenge. Moreover, you would likely experience anxiety about the test, particularly if you have the tendency to feel anxious in testing situations. These physiological and psychological factors can affect your performance in that test, which, in your specific situation, is one that measures intelligence.

Stress generally occurs as the organism senses an actual or perceived threat to its internal or external homeostasis (D. S. Goldstein and McEwen 2002). Physiologically, stress activates two distinct neurobiological pathways: the rapid sympathetic–adrenal–medullary axis (SAM), which leads to a release of noradrenaline and adrenaline from the adrenal medulla, and the slow hypothalamic–pituitary–adrenal axis (HPA), which leads to a release of glucocorticoids (cortisol in humans) from the adrenal cortex (Russell and Lightman 2019).

Acute stress can have facilitating and impairing effects on cognition, which are directly mediated by noradrenaline, glucocorticoids, or their interaction (Sandi 2013). For instance, acute stress exposure results in impaired memory retrieval but improved memory consolidation (Wolf et al. 2016). Mechanistically, cortisol mainly exerts its effects by binding to glucocorticoid receptors (GRs) and mineralocorticoid receptors (MRs) that are expressed in a variety of brain regions, such as the prefrontal cortex (PFC), hippocampus, and amygdala.

Executive functions, which refer to higher-order cognitive processes associated with the PFC and essential for planning and goal-directed behavior, are also susceptible to stress effects (Cristofori et al. 2019; Friedman et al. 2006; Shields et al. 2016; Sternberg 1985). The three fundamental executive functions are working memory, inhibition, and cognitive flexibility (Diamond 2013). Although these core executive functions are correlated with each other to some extent, they are distinct processes, each differentially related to variations in grey and white matter in various PFC regions (Miyake et al. 2000; Smolker et al. 2015). In a meta-analysis, it has been shown that stress impairs working memory, cognitive flexibility, and cognitive inhibition while facilitating response inhibition (Shields et al. 2016).

Given the central role of executive functions in supporting high-order cognition, it is not surprising that these processes are considered essential to intelligence (Diamond 2013; Ger and Roebers 2023; van Aken et al. 2016). Intelligence is a complex concept, and a precise definition remains elusive. However, it can broadly be understood as the capacity to learn from experience, using metacognitive processes, and to adapt to changing environmental demands (Sternberg 2018). Cattell (1941, 1943) developed one of the most prominent theories of intelligence, indicating that it can be divided into two factors: fluid intelligence (Gf) and crystalized intelligence (Gc). Gf refers to the experience-independent ability to solve problems and involves domains such as comprehending figural and semantic relations, abstracting, and inductive reasoning, and it is higher in young adults compared to older adults. In contrast, Gc refers to the experience-dependent comprehension of one’s own culture and involves domains such as verbal comprehension and social situational behaviors, and it is higher in older adults (Horn and Cattell 1967). Previous research has shown that some or all core executive functions exhibit correlations with either one or both of these factors in children (Brydges et al. 2012; Duan et al. 2010), young adults (Friedman et al. 2006), and adults (Salthouse 2014; van Aken et al. 2016). Ackerman et al. (2005) argue that working memory significantly correlates with measures of intelligence, and the development in children’s working memory capacity contributes to their improved Gf (Meiran and Shahar 2018; Tourva and Spanoudis 2020; Wilhelm and Oberauer 2006). Moreover, converging evidence demonstrates that overlapping fronto-parietal brain regions underpin both intelligence and executive functions (Clark et al. 2017; Pineda-Pardo et al. 2016; Roca et al. 2010). In addition, shared genetic influences have been shown to contribute to both domains (Engelhardt et al. 2016; Gustavson et al. 2022; Nikolašević et al. 2020). Furthermore, Spearman (1904) proposed the influential concept of a general factor (*g*), which is a common determinant of performance across diverse intelligence tasks (Jensen and Weng 1994). According to this theory, performance on any task that measures intelligence is determined by the combination of three components: *g*, task-specific abilities, and task-specific measurement errors.

A variety of intelligence tests have been developed to measure intelligence quotient (IQ) scores, such as the Intelligence Structure Test 2000R (I-S-T 2000 R; Liepmann et al. 2007), the Wechsler Adult Intelligence Scale (WAIS; Wechsler 2008), or the Raven Progressive Matrices (RPM; Raven 1938). Intelligence is a prominent predictor of various real-life outcomes such as education and job attainment, and IQ tests are regarded as the most reliable and standardized instruments for assessing the general cognitive abilities of individuals (Schmidt and Hunter 2004; Strenze 2007; van der Maas et al. 2014; Zisman and Ganzach 2022).

Intriguingly, intelligence may not be the only factor that affects performance in an IQ task. Even though it is the main cognitive determinant, psychological factors can also play a role. For instance, test anxiety has been shown to explain part of the variation in IQ tests, as anxiety negatively affected numerical intelligence and performance in a given math task (Ashcraft and Kirk 2001; Schillinger et al. 2018). Moreover, test anxiety undermines academic performance in undergraduate students, primarily through its cognitive dimension “worry” (Cassady and Johnson 2002; Rana and Mahmood 2010). In a meta-analysis, general intelligence and Gf were found to be negatively correlated with test anxiety (Ackerman and Heggestad 1997). Test anxiety has also been thought of as the component that mediates the negative correlation between Neuroticism and intelligence (Moutafi et al. 2006). One potential explanation for the negative impact of anxiety on intelligence task performance lies in its ability to disrupt working memory and attentional control (Eysenck et al. 2007; Eysenck et al. 2005; Mowbray 2012; Owens et al. 2014).

Even though it has been shown that acute stress can affect executive functions (among other cognitive functions) and executive functions contribute to the concept of intelligence, to our knowledge, there has not been a systematic investigation of the relationship between acute stress and intelligence outcome measures. In this study, we thus investigated whether acute stress exposure and test anxiety have an influence on performance in an IQ task. We hypothesized that (i.) acute stress impairs performance in an IQ task, (ii.) test anxiety impairs performance in an IQ task, and (iii.) the detrimental effect of acute stress on IQ performance would be greater among individuals with higher levels of test anxiety.

## 2. Materials and Methods

### 2.1. Participants

Sample size was determined a priori via G*Power 3.1 (Faul et al. 2009) with the goal to obtain sufficient power (1 − β = 0.8) assuming a medium effect size (*f* = 0.25) in a repeated measures analysis of variance (rANOVA) with two measurements (stress vs. control) at the standard error level (α = 0.05). The power analysis yielded a minimum requirement of 34 participants. For interpretability, we express sensitivity as equivalent between-subjects Cohen’s *d* while computing power with the repeated measures formula: $d_{z}=\frac{\sqrt{2}f}{\sqrt{1-\rho }}$. With our actual sample size (*n* = 40, after excluding three who withdrew prematurely) and an observed correlation between repeated measures of ρ = 0.80, power was high for large and medium-to-large effects (*d* > 0.5), adequate for medium effects (*d* = 0.5), and insufficient for small effects (*d* = 0.2; Table 1).

Participants were aged 18–34 years (23.38 ± 4.42 years; mean ± SD) with a Body Mass Index of 19–31 (24.05 ± 2.73; mean ± SD), and recruited via online advertisements, mailing lists, and university classes at Ruhr University Bochum. Exclusion criteria included an acute or past neurological, psychiatric, cardiovascular, or immunologic disease; current or past medical or psychological treatment; drug use; and female sex, due to hormonal influence on stress and associated changes in central nervous system functioning (Goldfarb et al. 2019; Kirschbaum et al. 1999; Merz and Wolf 2017). All participants had normal or corrected vision and received monetary compensation (10 €/hour, 40–50 € total) or course credit. Prior to testing, participants were informed about all study procedures and provided written informed consent. The study was conducted in accordance with the Declaration of Helsinki as approved by the psychological ethics committee of the Ruhr University Bochum.

### 2.2. Stress Induction and Assessment

The study followed a two-day crossover within-subject design, in which participants underwent the socially evaluated cold-pressor test (SECPT; Schwabe et al. 2008) on one day and a non-stressful control condition on another day (28 days later). The SECPT is a standardized stress protocol that reliably induces subjective and physiological stress. During the SECPT, participants must insert their hands into ice-cold water (0–4 °C) for a period of a maximum of 3 min (which is not known by the participants). Simultaneously, they are videotaped and observed by an additional, distanced experimenter. Importantly, these psychological components are essential for the efficiency of this method, as the physical pain from the ice-cold water alone is not sufficient to activate the HPA axis. In the control condition, they immerse their hand in warm water (35–37 °C) and are neither videotaped nor observed. The order of conditions was counterbalanced across participants.

We assessed subjective and physiological markers of stress. For subjective stress, we asked four questions addressing the adversity of the situation (difficulty, unpleasantness, stressfulness, and painfulness) on a scale ranging from 0 (“not at all”) to 100 (“very”). The physiological stress response was assessed via cortisol, as measured out of salivary samples using Salivettes (Sarstedt, Nümbrecht, Germany) and cardiovascular measurements collected at several time-points (see below). Saliva samples were stored at -20 °C until assay. Cortisol concentrations were extracted from the samples using a time-resolved fluorescence immunoassay (IBL, Hamburg, Germany) at the Genetic Psychology Lab of Ruhr University Bochum and reported in nanomoles per liter (nmol/l). Intra- and inter-assay coefficients of variation were below 9.3%.

### 2.3. Assessment of Test Anxiety

We used the short version of the German Test Anxiety Inventory (TAI-G; Wacker et al. 2008), which was developed based on the psychometric properties and factor structure of the original German TAI-G (Hodapp 1991). It includes 15 items, has a high internal consistency, and correlates at r = 0.98 with the original TAI-G. The questionnaire assesses the trait of how anxious individuals are when taking an exam or test, irrespective of the specific nature or contents of the test. It consists of four subscales (Emotionality, Worry, Lack of Confidence, and Interference), from which a total score can be obtained.

### 2.4. Assessment of Fluid Intelligence

We used the short form of the I-S-T 2000 R in its German version (Liepmann et al. 2007), which lasts 77 min in total. This form contains three submodules measuring verbal, numerical, and figural intelligence, respectively. Each submodule consists of three tasks with 20 items each, which increase in difficulty level, and for each task, a predefined time limit is set. By using the submodules, it is possible to obtain scores for verbal, numerical, and figural IQ, as well as a composite total IQ. A main reason for the selection of this test was that it allows repeated measures by providing parallel forms A and C, which differ in the specific items but are matched for difficulty. It is recommended to insert a break of 28 days before conducting the parallel forms A and C, which we conducted for each participant, with the order of forms being counterbalanced across participants and treatments.

### 2.5. Experimental Procedure

**Figure 1:**
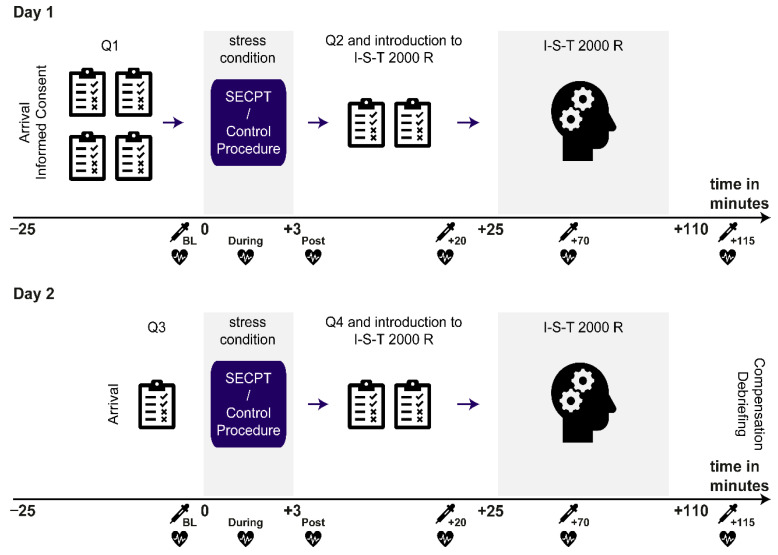
Experimental procedure. Participants were tested on two days with an intersession interval of 28 days. On one day, they underwent the SECPT, while on the other, a control procedure was conducted (with the order being counterbalanced across participants). Day one began with obtaining signed informed consent, before a series of questionnaires (Q1) was filled in. Then, the SECPT/control procedure took place, after which subjective stress assessment (Q2) and the introduction to the I-S-T 2000 R followed. About 25 min after stressor onset (or control procedure), the I-S-T 2000 R started, with a total net duration of 77 min. The procedure on day two was essentially the same, except for the reduced number of questionnaires at the beginning (Q3) and the respective other stress conditions as compared to day one. Numbers on the x-axes reflect time-points relative to stress (or control procedure) onset. 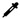 collection of saliva sample; 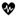 assessment of blood pressure, middle arterial pressure, and heart rate.

Testing sessions were performed between 12 p.m. and 6:30 p.m. to control for the circadian rhythm of cortisol secretion (Clow et al. 2004). On day one, upon arrival, participants awaited an acclimation period of 20 min, in which they read study information, gave written informed consent, and filled in a series of questionnaires. Then, participants underwent stress induction or control procedure. About 10 min after stress induction (or control procedure), participants were instructed on the I-S-T- 2000R, and the actual test started 25 min after stress induction (or control procedure). Physiological markers of stress were obtained at several time-points, while the subjective stress assessment was only performed immediately after stress induction (or control procedure). The testing session on day two followed four weeks later, and the procedure was essentially the same, except for the absence of already obtained questionnaires. Finally, participants were debriefed and compensated. The whole procedure is depicted in Figure 1.

### 2.6. Statistical Analysis

Statistical analyses were conducted in R 4.5.1 (R Core Team 2021) using the lme4 1.1-37 (Bates et al. 2015), lmerTest 3.1-3 (Kuznetsova et al. 2017), emmeans 1.11.2 (Lenth 2022), and brms 2.22.0 (Bürkner 2017, 2018, 2021) packages. Our first statistical analysis examined the success of stress induction by comparing physiological and subjective markers of stress between treatments. For cortisol concentrations and cardiovascular measures, we used rANOVAs with time and treatment as within-subject factors. Because cortisol concentrations typically exhibit a right-skewed distribution, we conducted a natural log (ln) transformation to obtain normally distributed data. In case of violation of the sphericity assumption, we used Greenhouse–Geisser adjustment and rounded the corrected degrees of freedom to the nearest whole number. Post-hoc pairwise comparisons were performed using Bonferroni-corrected *t*-tests. For the subjective markers, we used separate paired *t*-tests for all four measures (difficulty, unpleasantness, stressfulness, painfulness) as dependent variables, and the treatment was the independent variable.

To test our hypotheses postulating an impairing effect of cortisol and test-anxiety on the performance in an IQ-test, we built a linear mixed model with total IQ as the criterion, treatment (cortisol vs. placebo) as the within-subject predictor, and test-anxiety as the between-subject predictor. To investigate the meaningfulness of null findings, we additionally performed Bayesian statistics. In exploratory analyses, we built further linear mixed models with the subscales of IQ (verbal intelligence, numerical intelligence, or figural intelligence) as the criterion, respectively. In all linear mixed models, “subject” was added as the random factor and age, testing day, and sequence (to control for potential order effects) as covariates.

For all analyses, we centered age and TAI-G scores on the grand mean of all participants (Enders and Tofighi 2007). For the analysis of fixed effects, we used type III sum of squares. To estimate effect sizes, we used Partial Eta-Squared (η_p_^2^) for *F*-tests and Cohen’s d (*d*) for *t*-tests. Multicollinearity between predictors was not problematic (all variance inflation factors < 5). All statistical tests were conducted two-tailed at a significance level of α = 0.05.

## 3. Results

### 3.1. Increase in Physiological and Subjective Stress Markers After Stress Induction

**Figure 2:**
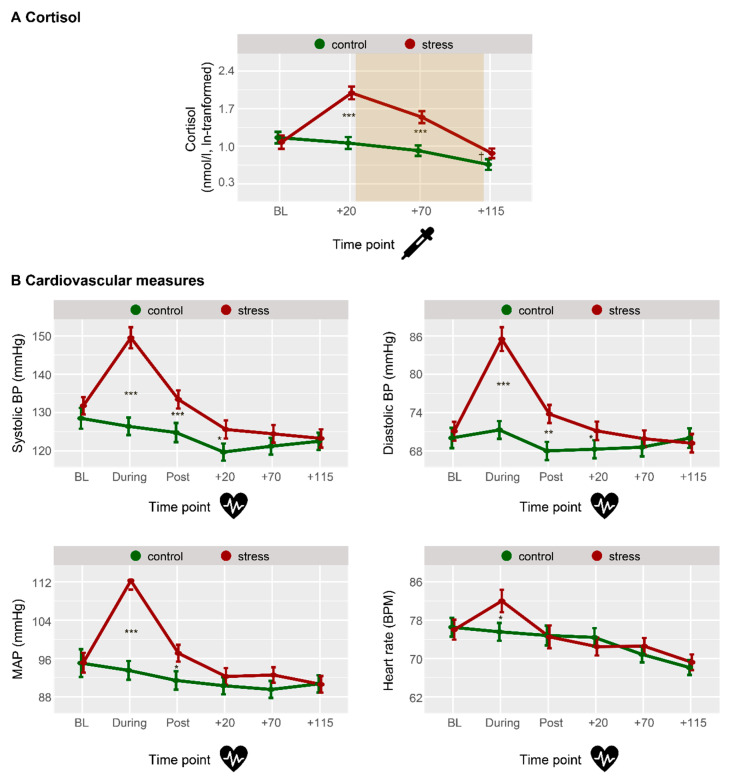
Time course of physiological stress measures. (**A**) Stress and control conditions did not differ for baseline cortisol concentration, but the stress condition induced higher cortisol at all time-points following stress induction. The shaded area represents the time window of the IQ test. (**B**) During the stress induction (and in some cases shortly after), the stress condition showed higher systolic and diastolic BP, higher MAP, and higher heart rate. Error bars represent SEM. BP: blood pressure, MAP: middle arterial pressure, *** *p* < .001, ** *p* < .01, * *p* < .05, ^†^ *p* < .10.

*Physiological stress.* For salivary cortisol concentrations, we found significant main effects of treatment (*F*_(1,38)_ = 28.46, *p* < .001, η_p_^2^ = 0.428, rANOVA) and time (*F*_(2,75)_ = 30.54, *p* < .001, η_p_^2^ = 0.446, rANOVA), and a significant interaction effect between treatment and time (*F*_(2,80)_ = 38.30, *p* < .001, η_p_^2^ = 0.502, rANOVA, Figure 2A). Post-hoc pairwise comparisons showed that the cortisol condition did not differ from the placebo condition during baseline (*t_(_*_39)_ = 0.75, *p_Bonferroni_ =* 1, *d* = 0.116, paired *t*-test), but exhibited higher cortisol concentrations for both time-points following the SECPT (both *t* ≤ −6.26, both *p_Bonferroni_ ≤* .001, both *d* ≤ −0.908, paired *t*-tests), while the last time-point still showed a trend for a difference (*t_(_*_39)_ = −2.57, *p_Bonferroni_ =* .056, *d* = −0.342, paired *t*-test). Analysis of cardiovascular measures complemented this picture, particularly by showing increased systolic and diastolic blood pressure, increased middle arterial pressure, and increased heart rate during the SECPT (all *t* ≤ −2.85, all *p_Bonferroni_ ≤* .042, all *d* ≤ −0.467, paired *t*-tests, Figure 2B).

*Subjective stress.* In the stress condition, participants judged the experimental procedure as more difficult, more unpleasant, more stressful, and more painful compared to the control condition (all *t* ≤ −9.22, all *p_Bonferroni_ <* .001, all *d* ≤ −1.822, paired *t*-tests, Table 2). The assessment of subjective stress markers thus complemented the picture of an overall successful stress induction.

### 3.2. Stress and Test Anxiety Do Not Affect IQ-Test Performance

To investigate the roles of stress and test anxiety in IQ-test performance, we incorporated these variables (along with the covariates day, sequence, and age) into several linear mixed models, with each model only differing by the specific outcome score used as the dependent variable (total IQ, figural IQ, numerical IQ, verbal IQ).

**Figure 3:**
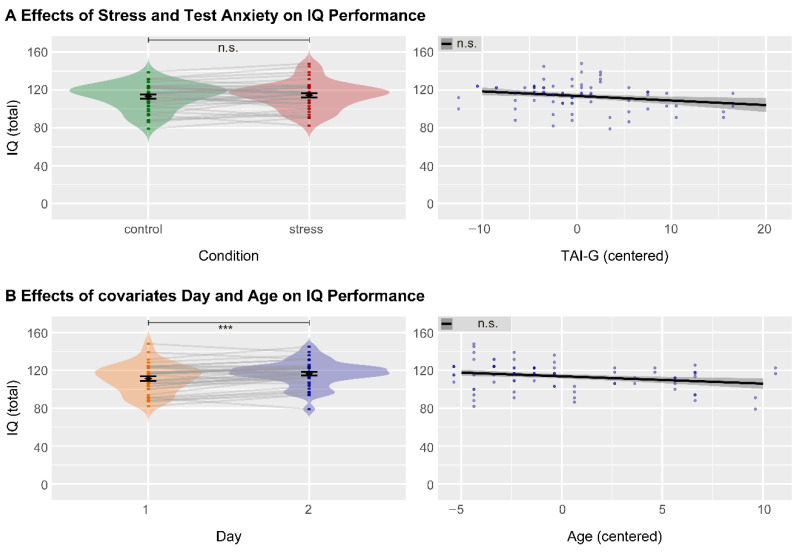
No effects of stress and test anxiety on total IQ performance. (**A**) Neither condition nor the test anxiety significantly affected total IQ performance. (**B**) From the covariates, day showed a significant effect on total IQ performance, indicating a higher IQ on the second day. IQ: intelligence quotient, TAI-G: test-anxiety inventory (German), *** *p* < .001, n.s.: not significant.

We observed no effects of stress (*F*_(1,35)_ = 0.74, *p =* .395, η_p_^2^ = 0.021, lmm, Figure 3A, left), test anxiety (*F*_(1,34)_ = 1.90, *p =* .178, η_p_^2^ = 0.053, lmm, Figure 3A, right) or their interaction (*F*_(1,35)_ = 0.13 *p =* .718, η_p_^2^ = 0.004, lmm) on total IQ performance (see also Table 3). From the covariates, only day showed a significant effect (*F*_(1,35)_ = 14.81, *p* < .001, η_p_^2^ = 0.297, lmm, Figure 3B, left), indicating increased total IQ performance on the second testing day.

**Figure 4:**
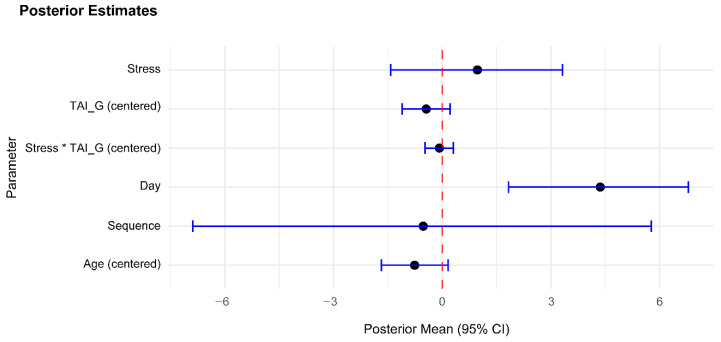
Forest Plot of Posterior Estimates. Posterior means for stress, test anxiety, and their interaction are close to zero, and their 95% credible intervals (horizontal bars) include zero, indicating that they do not exert an effect on total IQ performance. Day, on the other hand, influences total IQ performance, with a posterior mean of 4.36 and a 95% CI of [1.83, 6.79]. Data obtained from model comparisons between a full model including all parameters and reduced models, each missing the respective parameter.

Lastly, to assess the meaningfulness of the null findings of stress and test anxiety, we conducted equivalent Bayesian analyses (Figure 4), which provided very strong evidence for an absence of a stress effect (BF_10_ = 0.015), decisive evidence for an absence of an effect of test anxiety (BF_10_ = 0.008), and strong evidence for an absence of their interaction (BF_10_ = 0.044).

### 3.3. Exploratory Analysis: IQ Subscales

The analyses of the three subscales did not reveal further significant effects. Interestingly, day only exerted a significant effect in two out of the three subscales (numerical and verbal: both *F*_(1,35)_ ≥ 5.29, both *p ≤* .028, both η_p_^2^ ≥ 0.131, lmms), while it did not for figural IQ (*F*_(1,35)_ = 2.29, *p =* .139, η_p_^2^ = 0.061, lmm). This indicates that the general training effect from day one to day two for IQ performance is driven by training effects in numerical and verbal, but not in figural, IQ performance.

## 4. Discussion

The goal of this study was to test whether acute stress and test anxiety affect performance in an IQ test. To this end, we successfully induced stress with the SECPT, assessed participants’ trait test anxiety levels, and measured their performance on an established IQ test. We found no effects of stress or test anxiety on intelligence, and Bayesian analyses provided evidence for the absence of these effects. However, we found an effect of day, suggesting improvement in IQ test performance on the second testing day.

Previous studies showed that acute stress exposure influences executive functions (Fabio et al. 2022; Shields et al. 2016; Starcke and Brand 2016). More specifically, working memory, inhibition, and flexibility are generally negatively affected by acute stress. Given that executive functions are considered to be tightly associated with intelligence, we predicted worse performance in an intelligence test after stress exposure. Contrary to our expectation, stress did not impair performance in an IQ task. Generally, stress effects on cognition are highly variable, depending on many factors, including the timing between stressor onset and cognitive task. For instance, the heterogeneous effects of stress on working memory (Shields et al. 2016) are discussed in a recent model by Geißler et al. (2023), which posits that the discrepancies partly stem from variations in the time interval between stress induction and task administration. That is why we exploratively analyzed performance on each subtest separately. Here, we found no stress effects, neither on the verbal nor the numerical subtests, both of which were completed in the time window where cortisol levels were highly elevated after stress, nor on the figural subtest during which cortisol levels were not as strongly elevated. But because the different subtests measure different abilities, it would be enlightening to examine whether there is an interaction between timing and subtest, i.e., by counterbalancing the order in which subtests are undertaken. Moreover, future studies may consider employing abbreviated versions of intelligence tests, such as the short version of the Raven’s Advanced Progressive Matrices Test (APM; Arthur and Day 1994; Raven et al. 1985), to ensure that testing occurs completely within the time window of elevated cortisol levels.

Another moderator of stress effects is stress intensity. In the present study, participants underwent the SECPT, which is a widely used stress paradigm; however, it is less potent at eliciting mood disturbances and a stress response compared to the Trier Social Stress Test (Kirschbaum et al. 1993; Shields et al. 2017). Reduced stress intensity in our study may explain the lack of detrimental effects following stress exposure. In a previous study, TSST exposure impaired performance on an N-back task, whereas SECPT exposure had no effect (Giles et al. 2014). The role of stress intensity is directly related to the dose-dependency of cortisol effects (Joëls et al. 2018), which itself is related to the involved receptors, MRs and GRs. Under basal conditions, MRs are largely occupied due to their high affinity for cortisol, whereas GRs require elevated cortisol levels, such as those induced by stress exposure, for substantial activation (Reul and de Kloet 1985). In the current study, the moderately elevated cortisol levels following SECPT may not have been sufficient to elicit substantial GR activation. GR activation, however, is crucial for the effects of stress on executive functions, such as working memory and flexibility, as GRs modulate prefrontal cortex activity and top–down regulation (Barsegyan et al. 2010; Joëls and Baram 2009). However, several studies have shown impairments in cognitive or neural processes after the SECPT as well (e.g., Akan et al. 2023; Byrne et al. 2020; Langer et al. 2023; Liu et al. 2024; Pötzl et al. 2023; Zlomuzica et al. 2022).

Furthermore, although executive functions, particularly working memory, have been regarded as central to intelligence, they are not the only cognitive factors that determine IQ test performance. Participants’ working memory capacity may have been negatively affected by stress exposure without influencing IQ performance, potentially due to the recruitment of other compensatory mechanisms. Nonetheless, which specific cognitive functions contribute to intelligence remains a debated topic in the field, as there are also studies suggesting that the relationship between executive functions and intelligence is not straightforward. Although cognitive flexibility and inhibition are often associated with higher cognitive abilities, several studies suggest that these functions may not be linked to intelligence (Ardila 2000; Benedek et al. 2014; Friedman et al. 2006). Additionally, the role of working memory in intelligence is also questioned by studies showing that working memory trainings only provide short-term and domain-specific improvements, without leading to gains in general intelligence (Melby-Lervåg and Hulme 2013; Redick et al. 2015; Shipstead et al. 2012). From a practical perspective, it is reassuring that moderate acute stress does not have an impact on IQ performance, i.e., does not affect the validity in diagnosis settings.

In our second hypothesis, regardless of stress manipulation, we expected test anxiety to be associated with IQ test performance, which was not the case. According to the Attentional Control Theory (Eysenck et al. 2007), anxiety disrupts the balance between bottom–up and top–down processing of attention and thereby negatively affects cognitive performance, especially when the attentional demands of the task are higher (Derakshan and Eysenck 2009). However, although anxiety has adverse effects on the processing efficiency of the stimuli, it does not necessarily impair the performance significantly, as high-anxious individuals often compensate through increased effort, particularly when task demands are high (Eysenck and Derakshan 2011). This may explain why we did not observe negative effects of test anxiety on intelligence scores, as IQ tests are typically considered highly demanding and may elicit compensatory effort in anxious individuals. In line with this, it has been shown that high-anxious individuals exert more effort in tasks that require attentional control, as indicated by amplified neural activity, even though their performance was equal to that of low-anxious individuals (Righi et al. 2009; Savostyanov et al. 2009). Furthermore, the subjective importance attributed to a task can amplify anxiety levels even more (Nie et al. 2011). In line with this, the perceived relevance of the test might modulate the effects of anxiety on performance. Notably, studies reporting impairing effects of anxiety on cognitive performance have largely relied on its relationship with academic outcomes, which typically represent high-stakes situations for individuals. In our study, however, participants may not have perceived the test as consequential as a formal examination required for their degree, which is more relevant and has a more direct impact on their lives. As we did not assess perceived importance in our study, this possibility warrants further consideration in future studies. Moreover, a meta-analysis demonstrated that impaired working memory capacity mediates the relationship between anxiety and cognitive performance (Moran 2016). As our sample primarily consisted of university students, their working memory capacity is likely to be relatively high, given that higher education is positively associated with working memory capacity (Souza-Talarico et al. 2007). It is possible that participants’ high working memory capacity helped them counteract the influences of anxiety. Consistent with this reasoning, the relatively high IQ scores observed in our sample might explain the absence of stress and anxiety effects. Previous research shows that individuals with high intelligence have more available resources, enabling them to perform better in a given task (van der Meer et al. 2010).

Independent of stress exposure, participants had higher scores on day two even though they completed a different, parallel version of the test. The parallelism guaranteed that participants could not learn specific items, but it could not avoid a better understanding of the structure of the test, and this likely led to improved performance on the second day. This finding is in line with previous studies reporting increased scores upon retesting, although IQ scores are generally considered relatively stable over time (Catron and Thompson 1979; Deary 2014; Larsen et al. 2008; Lassiter and Matthews 1999). J. D. Matarazzo (1990) argued that IQ tests should not be regarded as a sole diagnostic tool, even though such tests demonstrate high test–retest reliability. Even with well-constructed alternate forms, retesting can yield both higher and lower scores. Overlooking the practice-related test–retest gains has led to misleading conclusions about medical intervention outcomes. Patients who received carotid endarterectomies exhibited improved IQ scores, but research showed that these improvements are mostly from practice effects (S. G. Goldstein et al. 1970; R. G. Matarazzo et al. 1979; Parker et al. 1983). Furthermore, some longitudinal studies examining the effects of aging on cognitive abilities failed to demonstrate declines in Gf scores, as practice effects obscured genuine age-related declines (Kaufman 2021; Lichtenberger and Kaufman 2009; Salthouse 2014). IQ scores also play a critical role in sensitive domains, highlighting the importance of rigorous investigation into practice-related changes. For example, in several U.S. states where capital punishment is still permitted, legal statutes require that defendants score above 70 on an IQ test, as lower scores denote intellectual disability and preclude execution (Cooke et al. 2015). Therefore, research employing IQ assessments should rigorously control for prior test exposure by excluding participants with previous experience, and similar caution should be exercised in clinical and diagnostic contexts.

Furthermore, we hypothesized an interaction between stress and test anxiety. Specifically, we expected that high-anxious participants would exhibit greater performance impairments following stress exposure. However, contrary to our expectations, no such interaction was observed. Given the absence of main effects for both stress exposure and test anxiety, the lack of significant interaction between the factors is not surprising. It is conceivable that a more potent stressor (i.e., TSST) or a different stress-timing, among other factors, could elicit performance impairments, particularly in highly anxious individuals.

### Limitations

A few limitations of this study need to be addressed. Our sample size was not large enough to detect small effects with sufficient power. Future studies are warranted that specifically aim at investigating small-sized effects with large sample sizes. Furthermore, we used a standardized laboratory stressor to induce a stress response. While this is typically conducted in stress research, certain real-life stressors can be more potent and complex and thus may exert additional and stronger influence. In the I-S-T 2000 R, participants have a specific time limitation to complete each subtest, and they are required to move on to the new subtest once the predefined time expires, regardless of whether they have completed the section. Notably, we did not record the amount of time participants needed to complete individual subtests. Thus, it remains possible that participants’ processing speed was influenced by stress, even though their accuracy was not. While processing speed is often not assessed on the item-level in the context of IQ testing, it is a common measure for specific cognitive abilities. Therefore, future studies might benefit from incorporating those measurements. Moreover, we used the TAI-G, which measures test anxiety as a trait; unfortunately, we lacked measures of state anxiety immediately before or during the test. It thus remains possible that participants with higher TAI-G scores did not experience heightened anxiety at testing. Lastly, we collected data only from males, and findings cannot be directly generalized to females, especially because gonadal hormones play a substantial role in stress reactivity (Jentsch et al. 2022), and females report higher levels of test anxiety (Chapell et al. 2005; Núñez-Peña et al. 2016). Future studies are warranted in which data from both sexes are collected.

## 5. Conclusions

We report that neither acute stress nor test anxiety affected performance in an IQ test, nor did they show an interaction effect. In contrast, Bayesian analyses indicated the absence of these effects. Acute stress, thus, might not be problematic for the assessment of intelligence in diagnostic settings. However, using two parallel versions of an intelligence test with an interval of four weeks revealed significantly increased IQ scores on the second testing day, indicating that repetitive testing influences outcome measures and thus should be interpreted with caution, both in scientific and diagnostic settings.

## Figures and Tables

**Table 1:** Power based on different effect sizes.

*d_z_*	*f*	Power (1 − β)
0.2	0.063	0.235
0.3	0.095	0.457
0.4	0.123	0.694
0.5	0.158	0.869
0.6	0.190	0.959
0.7	0.221	0.991
0.8	0.253	0.999

Note. *d_z_* represents the equivalent between-subjects Cohen’s *d* while computing power with the repeated measures formula: $d_{z}=\frac{\sqrt{2}f}{\sqrt{1-\rho }}$, using an observed correlation of repeated measures of ρ = 0.80, and where *f* represents classical Cohen’s *f.*

**Table 2:** Differences in subjective stress between conditions.

	Control Condition	Stress Condition	*p*
difficulty	1.25 ± 4.04	65.00 ± 25.12	<.001
unpleasantness	3.00 ± 13.44	49.75 ± 31.74	<.001
stressfulness	1.50 ± 4.27	44.75 ± 29.53	<.001
painfulness	0.75 ± 3.50	71.75 ± 23.95	<.001

Note. Values represent mean ± standard deviation on a numeric rating scale ranging from 0–100, *p*-values extracted from separate paired *t*-tests between conditions; data presented for final sample size of *n* = 40.

**Table 3:** Difference in IQ between conditions.

	Control Condition	Stress Condition	Cohen’s *d*
total IQ	113.20 ± 14.02	114.48 ± 14.66	0.09
numerical IQ	113.91 ± 15.13	115.04 ± 16.56	0.07
verbal IQ	109.56 ± 11.87	110.76 ± 11.27	0.10
figural IQ	106.26 ± 16.16	106.64 ± 14.53	0.02

Note. Values represent mean ± standard deviation; Cohen’s *d* values based on paired data; data presented for final sample size of *n* = 40.

## Data Availability

The dataset is available on request from the authors.
